# Isolevuglandins Scavenger Ameliorates Myocardial Ischemic Injury by Suppressing Oxidative Stress, Apoptosis, and Inflammation

**DOI:** 10.3389/fcell.2022.836035

**Published:** 2022-03-09

**Authors:** Junjie Guo, Fengqiang Xu, Hongwei Ji, Yajun Jing, Li Shen, Xinyu Weng, Longgang Hu

**Affiliations:** ^1^ Department of Cardiology, The Affiliated Hospital of Qingdao University, Qingdao, China; ^2^ Qingdao Municipal Key Laboratory of Hypertension (Key Laboratory of Cardiovascular Medicine), Qingdao, China; ^3^ Department of Critical Care Medicine, The Affiliated Hospital of Qingdao University, Qingdao, China; ^4^ Department of Cardiology, Zhongshan Hospital, Shanghai Institute of Cardiovascular Diseases, Fudan University, Shanghai, China; ^5^ Key Laboratory of Viral Heart Diseases, National Health Commission, Shanghai, China; ^6^ Key Laboratory of Viral Heart Diseases, Chinese Academy of Medical Sciences, Shanghai, China; ^7^ Department of Cardiology, The Affiliated Cardiovascular Hospital of Qingdao University, Qingdao, China

**Keywords:** myocadial infarction, oxidative stress, apoptosis, cardiac remodeling, inflammation, isolevuglandin scavenger

## Abstract

Augmented levels of reactive isolevuglandins (IsoLGs) are responsible for cardiovascular diseases. The role of IsoLGs in myocardial infarction (MI) remains elusive. Here we explored the effect of IsoLGs scavenger 2-hydroxybenzylamine (2-HOBA) in post-infarction cardiac repair. We observed that infarcted cardiac tissues expressed high IsoLGs in mice. Following MI injury, 2-HOBA treated mice displayed decreased infarction area and improved heart function compared with the saline-treated group. Moreover, 2-HOBA effectively attenuated MI-induced cardiac remodeling, oxidative stress, apoptosis, and inflammation. 4-hydroxybenzylamine (4-HOBA), a less reactive isomer of 2-HOBA, barely antagonized the MI-induced injury. These findings suggest that IsoLGs elimination may be helpful in MI therapy.

## Introduction

Myocardial infarction (MI) remains a life-threatening coronary heart disease worldwide (Seropian et al., 2014). Despite various current treatments, many patients with MI still develop heart failure due to loss of cardiomyocytes, suboptimal healing, and adverse cardiac remodeling (Seropian et al., 2014). Therefore, novel approaches urgently need to improve myocardial ischemia and optimize the infarct healing process after MI.

Cardiac remodeling is associated with the progression of heart failure and poor prognosis In patients with MI (Frangogiannis, 2012). Post-infarct healing and scar formation are critical in improving infarcted myocardium and preventing poor remodeling and heart rupture (Yan et al., 2017). Studies have consistently demonstrated that cardiac postinfarction repair is associated with elevated myocardial oxidative stress and cardiac inflammatory responses (Huang et al., 2020; Yang et al., 2017). Following MI, reactive oxygen species (ROS) are critical in the pathogenesis of cardiac remodeling, which is associated with impaired ventricular function. Oxidative stress at high levels leads to many injury-associated changes: pro-inflammatory cytokine release, myocyte apoptosis, cardiac fibrosis, and hypertrophy (Gilleron et al., 2009; Hilgendorf et al., 2014; Marinkovic et al., 2020; Yu et al., 2018).

Studies also show that cardiac repair after myocardial infarction requires a coordinated inflammatory response (Hilgendorf et al., 2014). Ischemic cardiac injury triggers a robust inflammatory cascade, initiated by the early inflammatory phase that involves immune cell infiltration and clearance of irreversibly damaged tissues, followed by a repair phase with inflammation resolution, angiogenesis, myofibroblast proliferation, extracellular matrix deposition, and scar formation (Prabhu and Frangogiannis, 2016). At the same time, inhibition of overactive and prolonged post-infarction inflammation is effective in attenuating the progression of MI-induced heart failure (Frangogiannis, 2014).

Reactive aldehydes are derived from the peroxidation of polyunsaturated fatty acids. Isolevulandins (IsoLGs), also known as isoketals, are a large family of 4-ketoaldehyde regio- and stereo-isomers initially characterized in the 1980s (Salomon and Miller, 1985). Among the isolevuglandins (IsoLGs), those with 1,4-dicarbonyl moiety revealed the highest reactivity (Salomon, 2005). IsoLGs have been shown to exert potent inflammatory effects in many cell types involved in cardiovascular diseases (Davies and Zhang, 2017). Increased formation of IsoLGs protein adducts has been found in various conditions associated with oxidative stress, such as ethanol-induced liver injury (Roychowdhury et al., 2009), hypertension, cardiac hypertrophy, and heart failure (Kirabo et al., 2014; Shang et al., 2019; Wu et al., 2016). In mice, IsoLG-modified cardiac proteins induce CD4^+^ T cell activation in response to cardiac pressure overload (Ngwenyama et al., 2021). But the role of isoLGs in the development of MI is unclear.

2-hydroxybenzylamine (2-HOBA) reacts with IsoLGs, acting as the scavenger of IsoLGs, about 1,000 times faster than the *ε*-amine of lysine, inhibit protein modification (Davies et al., 2006). 4-HOBA, an isomer of 2-HOBA, is a less effective scavenger of 4-oxoaldehydes (Zagol-Ikapitte et al., 2010). Here we studied the role of 2-HOBA in the left anterior descending artery (LAD) ligation-induced MI. We found that post-MI injury was associated with increased IsoLGs in cardiac muscles, and reduced IsoLGs content effectively attenuated the damage.

## Materials and Methods

### Animals

All procedures in this study were carried out in accordance with guidelines by the Animal Care and Use Committee of The Affiliated Hospital of Qingdao University, China. Ligation of the left anterior descending branch (LAD) or sham operation was performed on experimental animals as described previously (Gao et al., 2010). Male C57BL/6 mice, aged 8–10 weeks, were randomly assigned to either sham surgery or LAD ligation using a simple randomly assigned single sequence. Briefly, mice inhaled 2% isoflurane and were anesthetized with an isoflurane delivery system but without ventilation. Subsequently, a small piece of skin (1.2 cm) was cut into the left thorax, and the LAD was permanently ligated with 6–0 silk sutures. Sutures were made about 2,3 mm below the tip of the left auricle. Mice that died 24 h after surgery were excluded from the analysis. The sham animals underwent the same procedure without coronary artery ligation. After ligation, the mice were randomly divided into three groups, which were treated with IsoLGs scavger 2-HOBA, 4-hydroxybenzylamine (4-HOBA, a less reactive analog of 2-HOBA) and normal salines, respectively. Previous studies have shown that 1 g/L 2-HOBA in drinking water effectively attenuates the angiotensin II-induced increase in IsoLGs in mouse aorta and kidney tissues (Kirabo et al., 2014; Wu et al., 2016). In this study, as previously mentioned, 2-HOBA and 4-HOBA were added to drinking water at a dose of 1 g/L and replaced every 2,3 days using light-repellent water bottles. Mice were sacrificed two weeks after sham surgery or LAD ligation of CO2/cervical dislocation, and tissues were collected for analysis.

### Infarct Area Assesment

The area of myocardial infarction was determined by trichlorination staining with triphenyltetrazole. Briefly, the heart is immediately frozen and sliced into 1 mm thick slices. Subsequently, the sections were incubated with 1.5% 2,3,5-triphenyltetrazolium chloride (TTC; Sigma-Aldrich) solution and photographed. Left ventricular area and infarct size were measured by computerized planar measurement and comprehensive analysis of successive sections from each mouse was performed using ImageJ software (National Institutes of Health, Bethesda, MD, United States).

### ROS Measurement

To determine the ROS production, frozen heart sections (5 μm) were stained with dihydroethidium (DHE, 5 μmol/L, Sigma) in a dark humidified chamber at 37°C for 15 min. Then wash with PBS 3 times (5 min each) and cover sliders. The slides were observed by a confocal fluorescence microscope (Zeiss). Three visions were randomly selected from each slide. Quantifications were performed with ImageJ.

### Transthoracic Echocardiography

Cardiac function was assessed with a Vevo2100 Ultrasound system (FUJIFILM VisualSonics, Japan) at a specified time post-surgery. Mice were mildly anesthetized with 1%,2% isoflurane and fixed in a heated (37°C) ECG platform. Left ventricular diameter and thickness of septum wall and posterior wall at the end of systolic and diastolic stages were observed in long and short axial positions. Left ventricular fractional shortening (LVFS), left ventricular end-systolic diameter (LVIDS), left ventricular end-diastolic diameter (LVIDD), and left ventricular ejection fraction (LVEF) were calculated using the spherical formula as previously described.

### TUNEL

Cell Death Detection Kit (C1088, Beyotime) for cell apoptosis studies. Tissue was treated with proteinase K working solution for 15–30 min at 37°C, or cell permeability solution was added for 8 min; Rinse with PBS twice; TUNEL reaction mixture was prepared, and the treatment group was mixed with 50 μl TdT+450μl luciferin labeled dUTP solution; In the negative control group, only 50 μl luciferase labeled dUTP solution was added. In the positive control group, 100 μl DNase one was added first, and the reaction was carried out at room temperature for 30 min. The following steps were the same as those in the treatment group. After the slides were dried, 50 μl TUNEL reaction mixture was added to the specimens (only 50 μl luciferase labeled dUTP solution was added to the negative control group), and the slides or sealing film was covered, and the samples were incubated in a dark wet box at 37°C for 60 min. Rinse with PBS 3 times; Apoptotic cells can be counted by adding one drop of PBS under the fluorescence microscope.

### Western Blotting

Add an appropriate amount of concentrated SDS-PAGE protein loading buffer to the collected protein samples. Heat at 100°C or in a boiling water bath for 3–5 min to fully denature the protein. After cooling to room temperature, put the protein sample directly into the SDS-PAGE glue adding well. Transfer the protein to PVDF membrane. After the membrane transfer, immediately place the protein-membrane into the pre-prepared Western washing solution and rinse for 1,2 min to wash away the membrane transfer fluid on the membrane. The western sealing solution was added and slowly shaken on a shaker for 60 min at room temperature. Dilute primary antibody with Western primary antibody diluent in appropriate proportion according to the instructions of primary antibody. Collagen III (1:500, PA5-34787, Invitrogen, United States), Catalase (1:1,000, 14097S, Cell Signaling Technology, United States), GAPDH (1:1,000, 5174s, Cell Signaling Technology, United States), SOD1 (1:1,000, 37385S, Cell Signaling Technology, United States), 3-NT (1:500, ab53232, Abcam, United Kingdom), Bcl2 (1:1,000, 3498S, Cell Signaling Technology, United States), Bax (1:1,000, 14796S, Cell Signaling Technology, United States). The HRP-labeled secondary antibodies were diluted with Western secondary antibody diluent in appropriate proportions and incubated in a side shaking bed at room temperature for 1 h. Add Western washing solution, wash for 5–10 min. Repeat three times. If the background is dirty, the washing time can be appropriately extended, and the washing times can be increased. Hypersensitive ECL luminescence solution was used to detect the proteins.

### RNA Extraction and Quantitative Real-Time PCR

According to the manufacturer’s instructions, 1 ml of Trizol reagent for every 100 mg of fresh cardiac tissue, mince on ice with a sterile scalpel, and homogenize with a sterile homogenizer or other equipment. Tissue lysate was transferred to a 1.5 ml RNA-free EP tube. Set on ice for 5 min. Add 200 ul chloroform to each tube, mix thoroughly and leave on ice for 10 min to completely dissociate the nuclear protein complex. Centrifugation at 13,000 RPM at 4°C for 15 min. During this period, take a new EP tube, add 500 ul isopropyl alcohol, and precool on ice. After centrifugation, the upper water phase (about 500 ul) was transferred to the new EP tube. Let sit on ice and precipitate alcohol for 10 min. Centrifuge at 13,000 RPM for 10 min to remove the supernatant. The RNA was washed and precipitated once with 1 ml 75% ethanol. Centrifugation at 12000rpm for 5 min. Remove the supernatant and air dry or vacuum dry the RNA precipitation for 5–10 min. Dissolve the RNA in 30-50 ul DEPC-treated deionized water (add water to dissolve according to the amount of RNA precipitation). Spectrophotometric analysis was performed to determine sample concentration and purity and reverse transcription was performed. cDNA was amplified by RT-PCR using SYBR Green Mix. Primer pairs used in this study are as follow: TNF-α, (forward) 5′-CCT​GTA​GCC​CAC​GTC​GTA​G-3′ and (reverse) 5′-GGG​AGT​AGA​CAA​GGT​ACA​ACC​C-3*'*; IL-1β, (forward) 5′-GAA​ATG​CCA​CCT​TTT​GAC​AGT​G-3′ and (reverse) 5′-CTG​GAT​GCT​CTC​ATC​AGG​ACA-3*'*; MCP-1, (forward) 5′-ATC​CCA​ATG​AGT​AGG​CTG​GAG​AGC-3′ and (reverse) 5′-CAG​AAG​TGC​TTG​AGG​TGG​TTG​TG-3*'*.

### Immunofluorescence Staining

Frozen sections of myocardial tissue were fixed with 4% formaldehyde, and cells were permeated with 0.3% PBS within 5–15 min. After permeability, the cells were washed with PBS for 3 × 5 min. The cells were blocked with 5% BSA blocking solution for 30 min. The primary antibody was incubated at 4° overnight. CD45 (1: 100; ab10558; Abcam, United Kingdom), Mac2 (1:200, AB283654; Abcam, Cambridge, United Kingdom) The PBST was rinsed for 3 times, 5 min each time. The secondary antibody was incubated at room temperature to avoid light for 1 h. The PBST was rinsed three times, 5 min each time, and rinsed again with distilled water. A drop of DAPI sealing tablet was added, and the fluorescence microscope was used to observe the fluorescence.

### Immunostaining for IsoLGs

The embedded paraffin sections were sealed with 10% BOVINE serum albumin serum at room temperature for 1 h, and then incubated with anti-D11 single-chain antibodies (Kirabo et al., 2014). Quantification was performed using NIH ImageJ software. The staining was observed by incubation with anti-HRP labeling (Sigma) and incubated with a streptavidin-horseradish peroxidase kit (Pierce) for 20 min. The activity of peroxidase was detected by 3, 3-diaminobenzidine tetrachlor (DAB).

### Lipid Peroxidation (MDA) Assay

Lipid peroxidation (MDA) assay kit (Abcam, AB118970) was used to measure MDA levels in the heart according to the instructions. In brief, 10–20 mg of fresh left ventricular tissue is homogenized in a solution containing butylhydroxytoluene. Centrifuge to remove insoluble parts and supernatant for analysis. The supernatant was mixed with a thiobarbituric acid (TBA) solution recombined in glacial acetic acid and incubated at 95°C for 60 min. The supernatant containing MDA-TBA adduct was added to a 96-well plate for analysis. The absorbance at 532 nm was measured with a microplate meter (Ito et al., 2021).

### Statistical Analysis

All data were expressed as mean ± SEM. *χ*2 test and Pearson correlation analysis were used to evaluate discrete variables and correlations. Multiple sets of data were compared using one-way ANOVA or Kruskal–Wallis test, followed by the Bonferroni post hoc test. *p*-value less than 0.05 was considered statistically significant. Statistical analysis was performed using GraphPad Prism 8.0 (Graph Pad Prism Software Inc., San Diego, CA).

## Results

### IsoLGs Scavenger 2-HOBA Strikingly Reduced the Increase of IsoLGs in MI-Induced Myocardial Tissue

**FIGURE 1:**
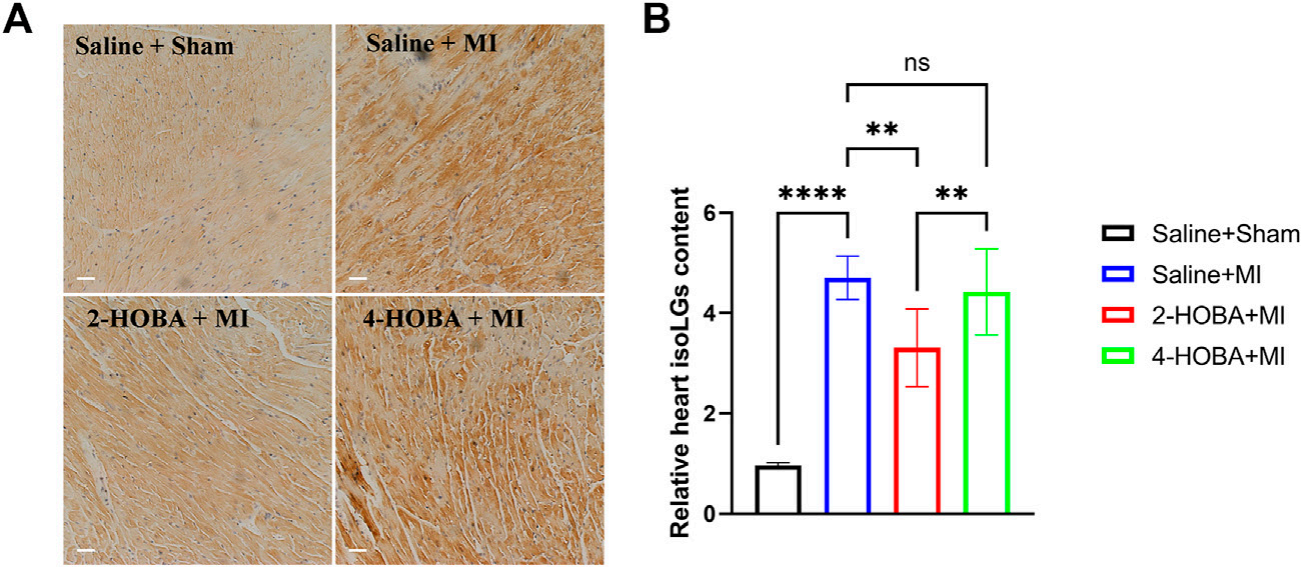
MI stimulates IsoLGs accumulation in heart tissues. **(A)**, Immunohistochemistry of cardiac sections showing accumulation of IsoLGs using a single-chain antibody that recognizes IsoLGs-lysine adducts on all proteins. Scale bars: 50 μm. **(B)** Quantification of IsoLGs in cardiac muscles (*n* = 7). **p < 0.01, ****p < 0.0001; ns, non-significant. All values are mean ± SEM.

After Left anterior descending artery (LAD) ligation, the levels of IsoLGs protein augmentation in the heart of MI mice were primarily detected by immunohistochemical staining. We have shown that 2-HOBA and 4-HOBA showed comparable effects with saline on cardiac function and morphology in the sham surgery group (Shang et al., 2019), so we only study the role of HOBA under MI surgery conditions. We found that IsoLGs protein level was significantly increased in cardiac tissues of mice with MI injury (Figures 1A,B). Accordingly, we found that the IsoLGs scavenger 2-HOBA effectively attenuated MI-induced increases of IsoLGs protein adducts in cardiac tissues in mice. However, 4-HOBA resulted in a bit of reduction of IsoLGs protein adducts compared to 2-HOBA, and the accumulation of IsoLGs was similar to that of control groups (Figures 1A,B). Our findings revealed the potential role of 2-HOBA in MI remission.

### 2-HOBA Attenuates MI Induced Cardiac Injury

**FIGURE 2:**
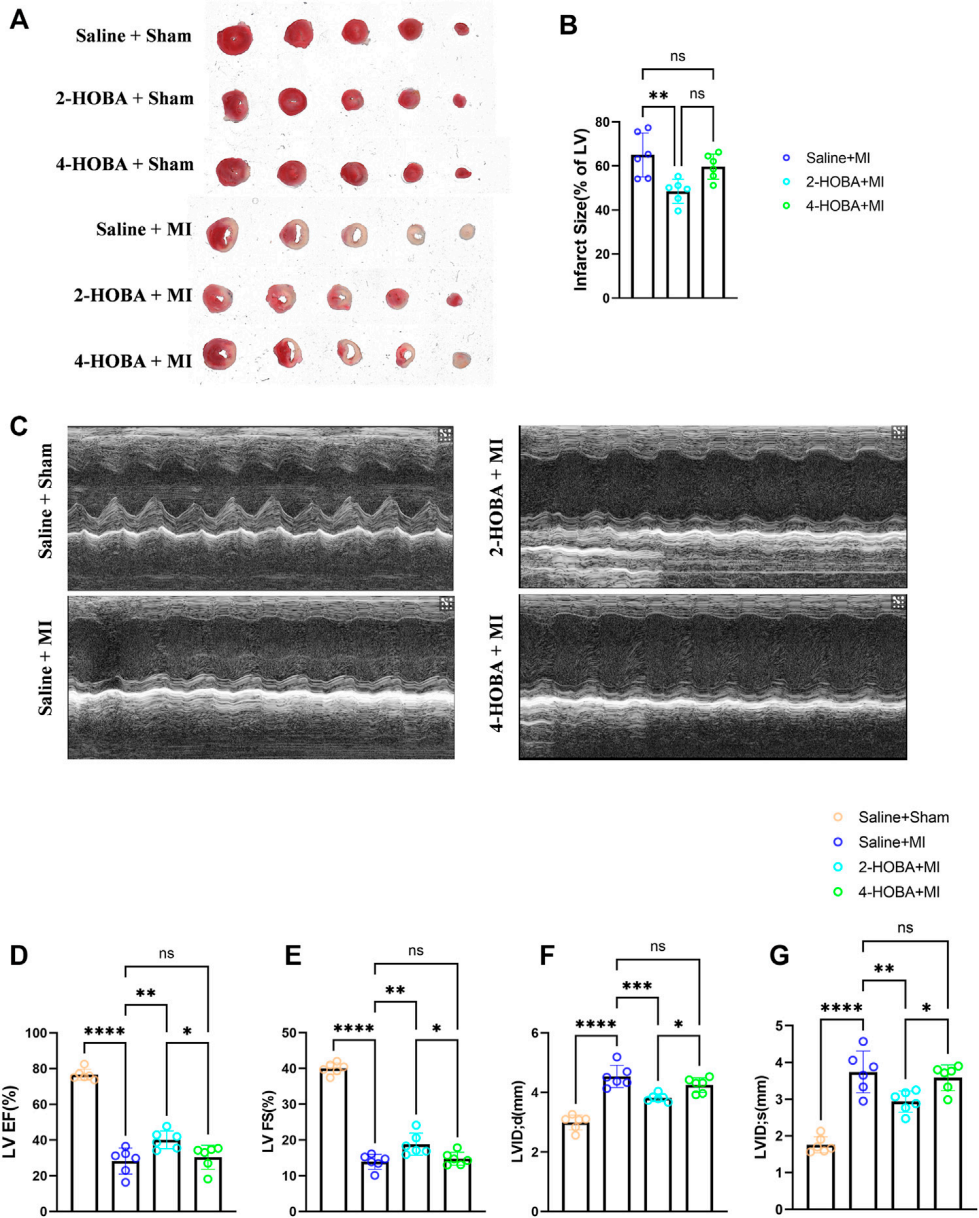
2-HOBA attenuates MI-induced cardiac injury. **(A–G)**, C57BL/6 treated with vehicle, 2-HOBA or 4-HOBA were subjected to left anterior descending artery (LAD) ligation or the sham procedure as described in Methods. **(A)**, Frozen sections were stained with triphe-nyltetrazolium chloride (TTC). **(B)**, Quantification of the infarct area (*n* = 6). **(C)**, Heart function was evaluated by echocardiography (*n* = 6). EF, Ejection fraction, FS, fractional shortening, LVID, left ventricular internal dimension, LVIDS, left ventricular internal dimension systole. *p < 0.05, **p < 0.01, ***p < 0.001, ****p < 0.0001; ns, non-significant. All values are mean ± SEM.

Next, we hypothesized that 2-HOBA might exert functional effects on MI. C57BL/6 mice treated with vehicle, 2-HOBA, or 4-HOBA were subjected to MI modeling. As anticipated, MI resulted in a significant increase in infarct size (Figures 2A,B) and a noticeable decline in cardiac function, as denoted by decreased left ventricular fractional shortening (LVFS) and left ventricular ejection fraction (LVEF) and increased left ventricular internal dimension systole (LVIDS) and left ventricular internal dimension (LVID), which were rescued by 2-HOBA administration (Figures 2C–G). 4-HOBA, however, did not afford noticeable improvements in heart function both 2-HOBA and 4-HOBA show comparable effects with saline on sham surgery condition as showed in Figure 2A. Overall, 2-hoba played a protective role in myocardial infarction.

### 2-HOBA Attenuates Post-MI Cardiac Remodeling

**FIGURE 3:**
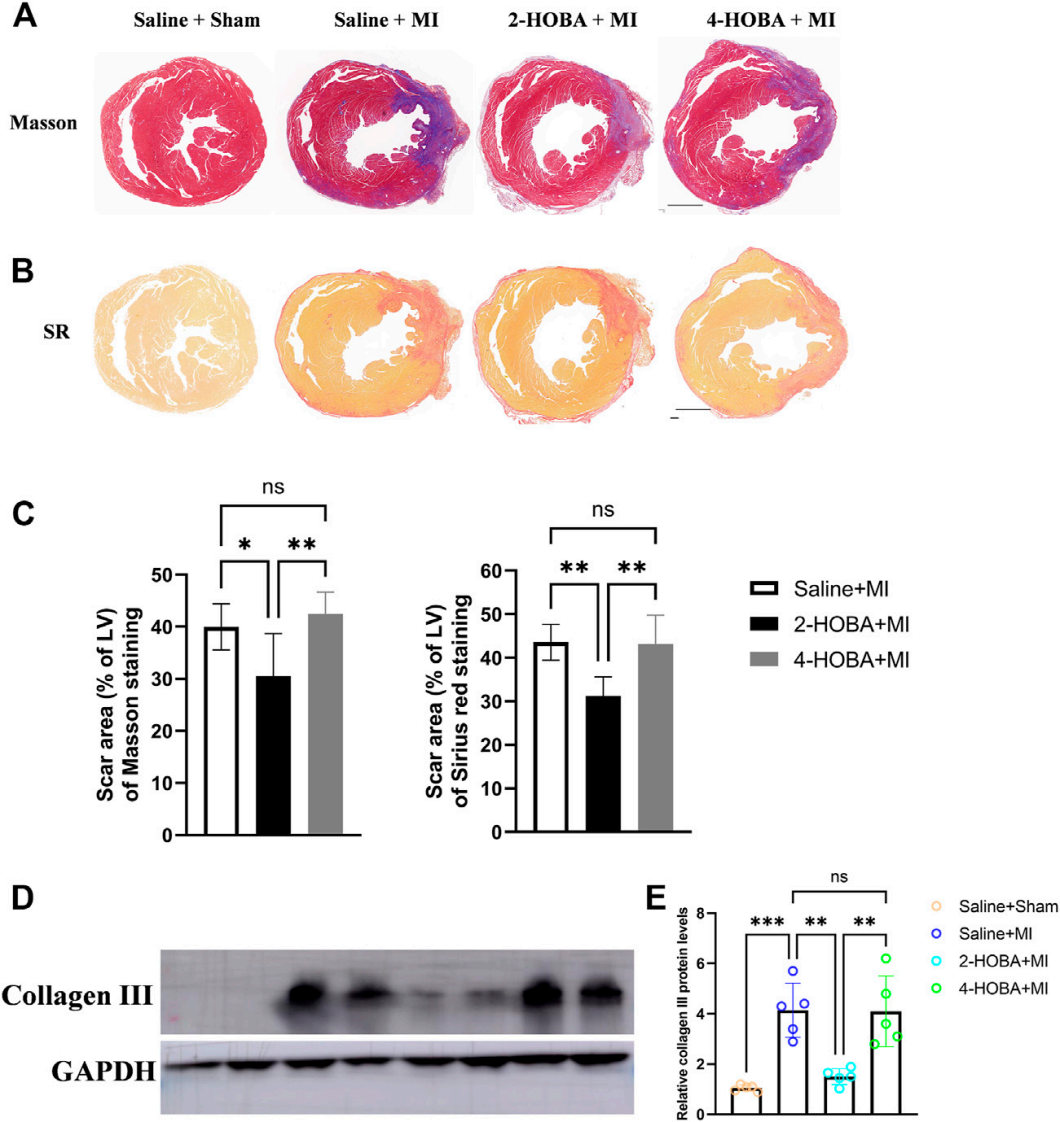
2-HOBA attenuates MI-induced cardiac remodeling. **(A–E)**, C57BL/6 treated with vehicle, 2-HOBA or 4-HOBA were subjected to left anterior descending artery (LAD) ligation or the sham procedure as described in Methods. **(A)**, Masson’s trichrome (TC) and Picrosirius red (PSR) staining of heart transverse sections to detect scar area after MI. Scale bars: 1 mm. **(B,C)**, Quantification of scar area, PSR (*n* = 6), TC (*n* = 6). **(D)**, Protein level of collagen III. **(E)**, Quantified data of immunoblotting band intensity (*n* = 5), *p < 0.05, **p < 0.01, ***p < 0.001; ns, non-significant. All values are mean ± SEM.

Further, Masson’s trichrome staining and Sirius red staining of infarcted cardiac muscle revealed increased scar area at the infarct area compared with those observed in sham mice, which were rescued by 2-HOBA treatment (Figures 3A–C). Moreover, western blot analysis of collagen III, one of the factors for cardiac fibrosis, also showed elevated expression in MI groups, which was reduced by 2-HOBA administration (Figures 3D,E). Cardiac remodeling was similar between MI and 4-HOBA treated MI groups. Taken together, our data confirmed the beneficial effects of 2-HOBA on post-MI cardiac remodeling.

### 2-HOBA Attenuates MI Induced Cardiac Oxidative Stress

**FIGURE 4:**
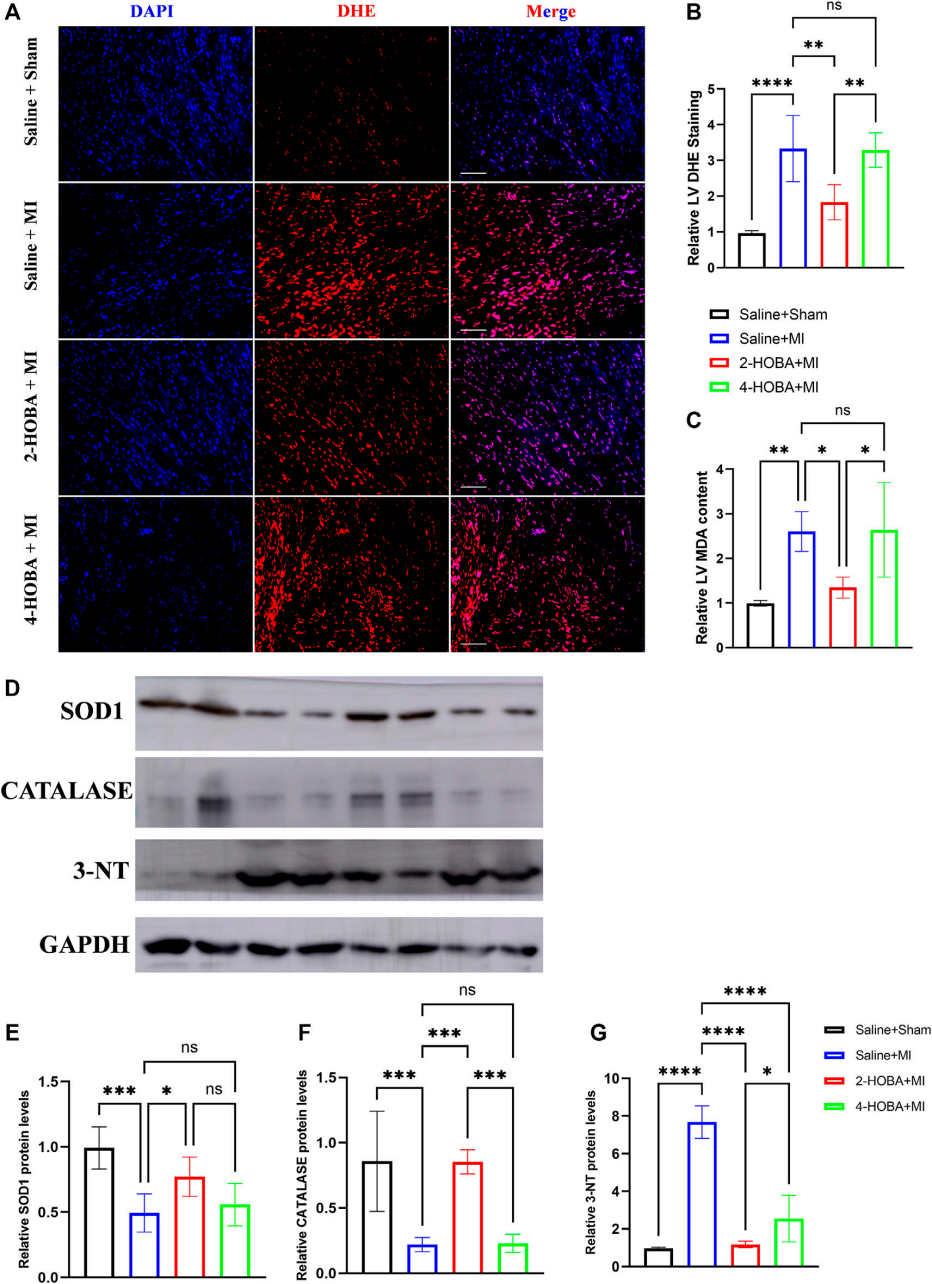
2-HOBA attenuates MI-induced cardiac oxidative stress. **(A–G)**, C57BL/6 treated with vehicle, 2-HOBA or 4-HOBA were subjected to left anterior descending artery (LAD) ligation or the sham procedure as described in Methods. **(A)**, Frozen sections were stained with dihydroethidium (DHE). Scale bars: 50 μm. **(B)**, Quantification of DHE positive area (*n* = 5), **(C)**, Quantification of MDA levels (*n* = 5). **(D)**, Representative immunoblot images of SOD1, Catalase and 3-NT. **(E–G)**, quantification of immunoblotting band intensity (*n* = 5). *p < 0.05, **p < 0.01, ***p < 0.001; ns, non-significant. All values are mean ± SEM.

Myocardial infarction (MI) is a life-threatening disease associated with a state of excessive oxidative stress that severely disrupts myocardial cell membranes and subcellular structures (Khalifa et al., 2021). DHE staining was used to analyze the ROS production in cardiac sections. As expected, reduced ROS production was observed in 2-HOBA treated hearts compared to those treated with vehicles post-MI (Figures 4A,B). Increased level of oxidative stress marker such as malondialdehyde (MDA) was also suppressed by 2-HOBA administration post-MI (Figure 4C). Accordingly, immunoblot analysis showed significantly increased antioxidant molecules superoxide dismutase 1 (SOD1) and Catalase. Still, it decreased expression of oxidant molecule 3-NT in myocardium lysates harvested from peri-infarct areas of 2-HOBA treated mice, compared with that of sham groups after MI (Figures 4D–G). Collectively, our data demonstrated that 2-HOBA improved cardiac function partly by reactive oxygen species clearance.

### 2-HOBA Attenuates MI Induced Cardiomyocytes Apoptosis and Inflammation

**FIGURE 5:**
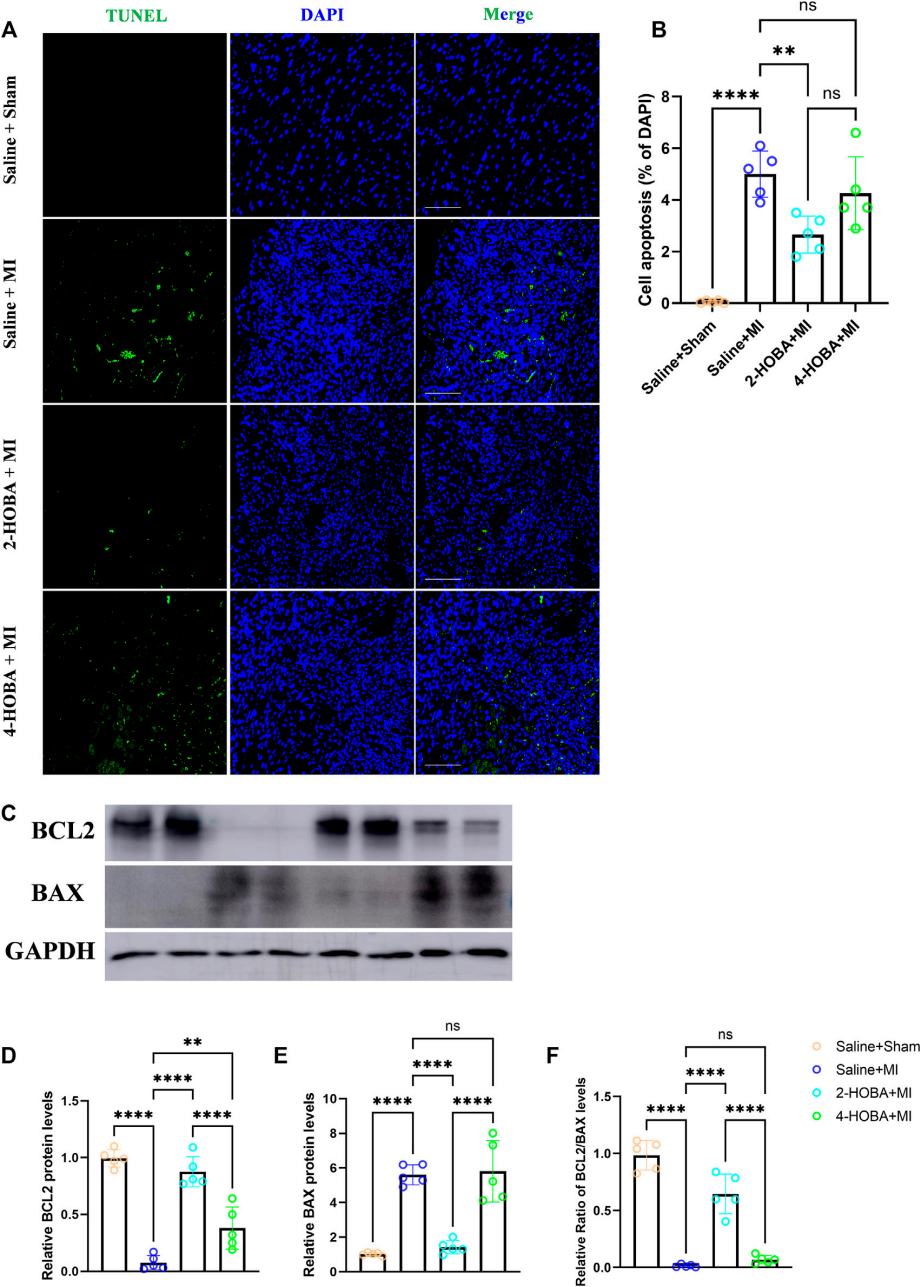
2-HOBA attenuates MI-induced cardiomyocytes apoptosis. **(A–F)**, C57BL/6 treated with vehicle, 2-HOBA or 4-HOBA, respectively, were subjected to left anterior descending artery (LAD) ligation or the sham procedure as described in Methods. **(A)**, *In situ* TUNEL staining of border areas of MI-operated hearts as well as from sham controls. Scale bars: 100 μm. **(B)**, Quantification of apoptosis cells (*n* = 5). **(C)**, expression of apoptosis-related proteins including BCL-2 and Bax in myocardial tissue lysates harvested from peri-infarct areas of post-MI hearts. **(F)**, Quantified data of immunoblotting band intensity (*n* = 5). **p < 0.01, ****p < 0.0001; ns, non-significant. All values are mean ± SEM.

**FIGURE 6:**
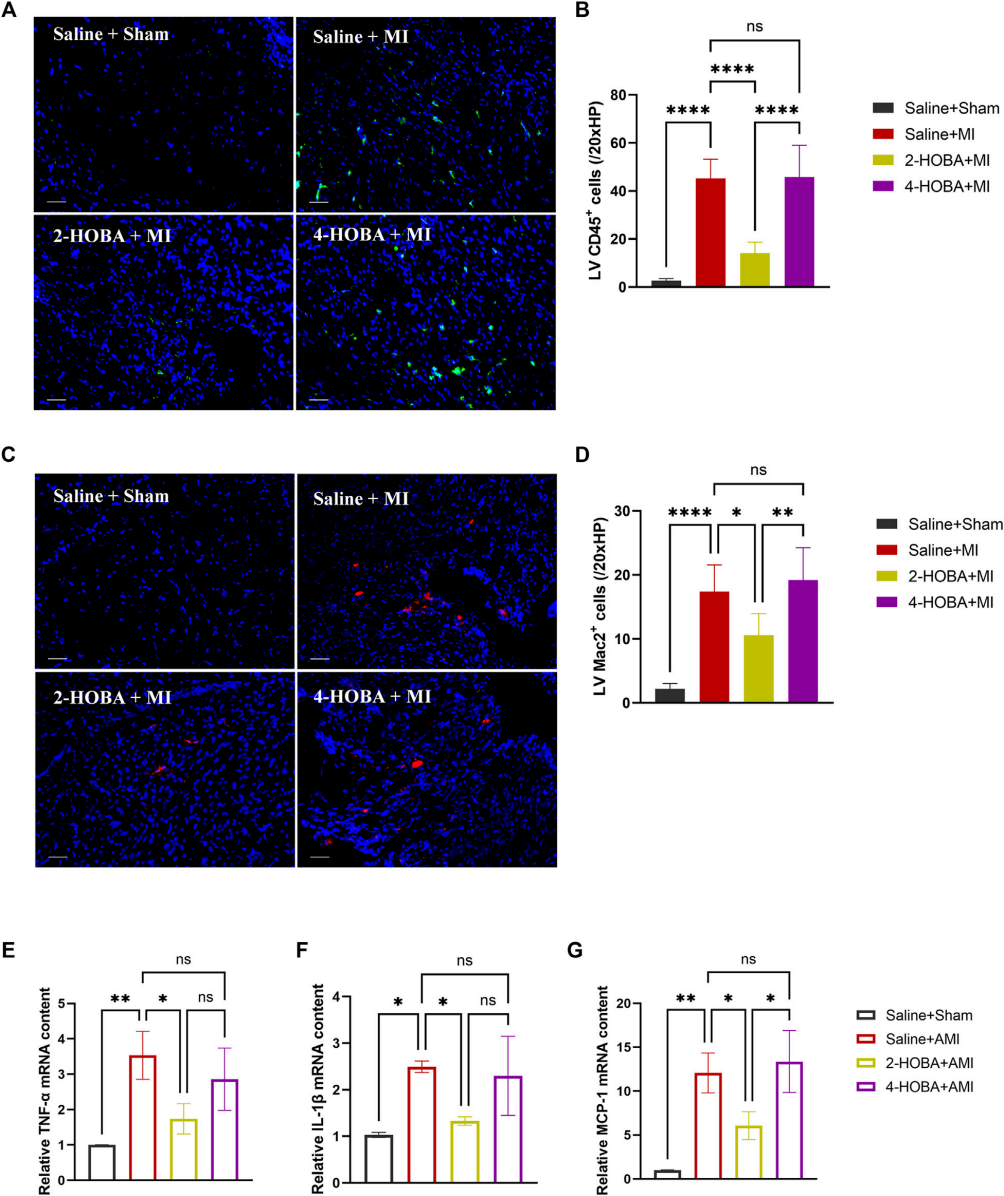
2-HOBA alleviates cardiac inflammation after AMI. **(A–G)**, C57BL/6 treated with vehicle, 2-HOBA or 4-HOBA, respectively, were subjected to left anterior descending artery (LAD) ligation or the sham procedure as described in Methods. **(A–D)**, Immunofluorescence staining and quantification of CD45 and Mac2 were performed on infarcts (*n* = 5). Scale bars: 50 μm. **(E–G)**, mRNA expression levels of major pro-inflammatory genes including interleukin (IL-1β), tumor necrosis factor (TNF-α), and monocyte chemotactic protein-1 (MCP-1) in infarcts, *n* = 3 per group. *p < 0.05, **p < 0.01, ****p < 0.0001; ns, non-significant. All values are mean ± SEM.

Evidence from previous studies showed that excessively activated inflammatory response and apoptosis are both crucial mechanisms contributing to cardiac ischemic injury (Abbate et al., 2008; Frangogiannis, 2014). Thus, TUNEL assay was performed to detect apoptosis in cardiac muscle after MI. In comparison with sham groups, a significant increase of TUNEL positive cells was observed in the border area of MI heart. 2-HOBA instead of 4-HOBA treatment significantly attenuated the MI-induced cardiomyocytes apoptosis (Figures 5A,B). Following this finding, immunoblot analysis revealed decreased considerably expression of pro-apoptosis molecule Bax. Still, increased anti-apoptosis molecule Bcl-2 in myocardium lysates harvested from peri-infarct areas of 2-HOBA treated mice, compared with that of sham groups after MI (Figures 5C–F). We next investigated whether 2-HOBA treatment impacts primary inflammatory response after MI. As expected, immunofluorescence staining of infarcted cardiac muscle detected reduced CD45^+^ and Mac2^+^ cells in the infarct area of the 2-HOBA mice (Figures 6A–D). Moreover, downregulation of major pro-inflammatory cytokines including interleukin (IL-1β), tumor necrosis factor (TNF-α), and monocyte chemotactic protein-1 (MCP-1) were observed in 2-HOBA treated hearts post MI, reflecting attenuated inflammatory response in MI tissue by 2-HOBA intervention (Figures 6E–G). 4-HOBA treatment did not afford detectable mitigation of apoptosis and inflammation. Taken together, 2-HOBA exerts protective effects on ischemic myocardial injury by attenuating apoptosis and reducing the production of pro-inflammatory mediators. These findings align with the observation that 2-HOBA treated mice subjected to MI exhibited modestly reduced scar area (Figures 3A–C).

## Discussion

Oxidative stress-induced lipid peroxidation is involved in the pathogenesis of several cardiovascular diseases, including myocardial infarction (MI), leading to cardiac dysfunction, acute cardiac arrest, and death. The main new finding was that myocardial infarction injury was associated with increased IsoLGs protein adducts in mice heart tissue (Figure 1). Pharmacological IsoLGs clearance 2-HOBA significantly reduced the rise in IsoLGs, cardiac remodeling and dysfunction, and cardiac inflammation and apoptosis in LAD ligation-induced tissues. In addition, we found that 4-HOBA, the isomer of antioxidant 2-HOBA, had little effect on reducing cardiac remodeling and dysfunction after LAD ligation, possibly due to its poor impact on reducing tissue IsoLGs protein adducts compared with 2-HOBA. These data suggest that IsoLGs protein adduct can be used as a marker of cardiac remodeling during myocardial infarction injury. The beneficial effect of IsoLGs scavenger 2-HOBA on cardiac repair is partly due to its antioxidant effect in this model.

Excessive inflammation and inefficient repair are worse outcomes after MI(Prabhu and Frangogiannis, 2016). For instance, suboptimal infarct healing contributes to progressive thinning and expansion of the infarct wall, resulting in a malformed scar, a high risk of cardiac rupture, and maladaptive cardiac remodeling (Jia et al., 2019). Consistent with this concept, we show that 2-HOBA treatment exhibited modestly reduced infarct size, improved heart function, and ameliorated adverse remodeling such as LV enlargement after MI (Figures 2, 3).

Oxidative stress occurs when oxidative metabolites exert toxic effects because of their increased production or altered cellular protective mechanisms. The heart needs oxygen but is also vulnerable to oxidative stress, especially when the heart is in ischemia, myocardial infarction, and other conditions. Oxidative stress accentuates myocardial apoptosis and inflammation, aggravating ischemic injury (Abbate et al., 2008; Chiong et al., 2011; Frangogiannis, 2014; Jia et al., 2019; Liu et al., 2017). Here, we demonstrate that 2-HOBA, instead of 4-HOBA, could efficiently inhibit myocardium oxidative stress (Figure 4) and exert protective effects on ischemic cardiac injury by inhibiting apoptosis in the peri-infarct area and limiting local ischemia-provoked inflammatory responses (Figures 5, 6).

Increased oxidative stress has been shown to contribute to post-infarction injury (Shilo et al., 2021). During lipid peroxidation, highly reactive dicarbonyl compounds are formed, including 4-oxononenal (4-ONE), 4-hydroxynonenal (4-HNE) malondialdehyde (MDA), and isoelemic acid lipids (IsoLGs). These reactive lipid dicarbonyl compounds bind to DNA, proteins, and phospholipids, leading to alterations in lipoprotein and cellular functions. In particular, modification of active lipid dicarbonyl species promotes inflammatory responses and toxicity associated with cardiovascular disease. y-ketoaldehydes, including isoketones, isoprenoids, and neuroketones, are highly reactive and vigorously promote inflammation (Davies and Zhang, 2017; May-Zhang et al., 2021). 2-HOBA could inhibit the formation of IsoLGs protein augmentation by reducing the overall tissue oxidative stress (Brame et al., 2004; Davies et al., 2004; Kirabo et al., 2014). It is reported that overexpression of NADPH oxidase subunit P22phox in mouse smooth muscle or increased oxidative stress induced by vascular-specific loss of extracellular SOD can increase vascular IsoLGs protein adducts. At the same time, superoxide scaverant Tempol, or IsoLGs scaverant 2-HOBA, significantly reduced the IsoLGs content in mouse blood vessels, prevented vascular inflammation, aortic sclerosis and hypertension, and prevented dendritic cells and T cell activation (Wu et al., 2016).

Improved myocardial ischemic injury is closely related to decreased cardiac oxidative stresses by inhibition of NADPH oxidases (Yu et al., 2018), inhibition of xanthine oxidoreductase (Berry and Hare, 2004), reducing of NOS uncoupling (Granger and Kvietys, 2015), or directly overexpressing various antioxidants.2-HOBA significantly attenuated TAC-induced LV ROS production (Shang et al., 2019) and AS progression (Tao et al., 2020), consistent with our findings. Nevertheless, the results demonstrate an essential role of IsoLGs in MI development.

### Limitations

The present study has several limitations. Here we first firmed the protective role of 2-HOBA in MI injury, evidenced by improved cardiac remodeling, alleviated cardiomyocyte apoptosis, mitigated inflammation, and reduced ROS production. However, the potential mechanisms are still elusive and need further study. Additionally, male mice were used in our experiments for the sake of less hormone variability. The outcome in female mice may not be identical to these findings. Further, more time points are required to study the effects of 2-HOBA on different stages of MI. Nevertheless, the results demonstrate an essential role of 2-HOBA in MI improvements.

## Data Availability

The original contributions presented in the study are included in the article. Further inquiries can be directed to the corresponding authors.
